# Automated enzymatic determination of plasma free fatty acids by centrifugal analysis

**DOI:** 10.1155/S1463924684000286

**Published:** 1984

**Authors:** D. P. Knox, D. G. Jones

**Affiliations:** Moredun Research Institute 408 Gilmerton Road Edinburgh EH17 7JH UK


Journal of Automatic Chemistry, Volume 6, Number 3 (July–September 1984), pages 152-154

### Automated enzymatic determination of plasma free fatty acids by centrifugal analysis

**D. P. Knox and D. G. Jones**

*Moredun Research Institute, 408 Gilmerton Road, Edinburgh EH17 7JH, UK*

### Introduction

Simple and specific measurement of free fatty acid (FFA) levels in plasma and serum is of practical value to the clinical biochemist and, in the authors' laboratory, in veterinary nutritional and metabolic studies. Most early methods, such as that of Duncombe [1], relied on the colorimetric determination of metal-FFA complexes in the organic phase of solvent extracted plasma. These techniques are relatively non-specific and, in addition, are extremely laborious and time-consuming.

Recently, alternative assays for FFA, based on specific enzymic oxidation by acyl-coenzyme-A-synthetase (ACS) and acyl-coenzyme-A-oxidase (ACOD), have been developed (Shimizu *et al.* [2]). Hydrogen peroxide generated by these reactions can be quantitated colorimetrically (Mizuno *et al.* [3]; Shimizu *et al.* [4]; Matsubara *et al.* [5]) and is directly related to the FFA content of the test sample. One such procedure involving specific peroxidase (POD)-dependent quinone-dye formation is now available commercially.

This article reports on the adaptation of the method for routine automated determination of FFA using an I.L. Multistat III microcentrifugal analyser.

### Materials and methods

### *Reagents and instrumentation*

Wako (Wako Pure Chemical Industries Ltd, Japan) NEFA C-Test (ACS-ACOD method) kits code No. 273-75409 were supplied by Alpha Laboratories, Eastleigh, Hampshire, UK.

A Cecil CE 595 double-beam spectrophotometer (Cecil Instruments Ltd, Cambridge, UK) was used for all manual determinations. Automated analyses were performed using an I.L. Multistat III fluorescence/light scattering microcentrifugal analyser (MCA) (Instrumentation Laboratory [UK] Ltd, Warrington, Cheshire). A solution of 152·3 mg oleic acid (Sigma Chemicals, Poole, Dorset, UK) in 100 ml 0·1% Triton X-100 (5 mmol oleic acid per litre) acted as a stock standard.

### *Samples*

Ovine and bovine whole-blood samples were collected in 10 ml Vacutainers (Becton-Dickinson [UK] Ltd, London) containing heparin (0·1–0·2 mg/ml blood), oxalate/fluoride (1·25 and 1·0 mg/ml blood respectively), EDTA (1 mg/ml) or no anticoagulant. Plasma or serum were removed following centrifugation at 5000 g for 10 min at 4°C.

### *Manual FFA determination*

FFA were estimated colorimetrically by the method of Duncombe [1] based on the extraction procedure proposed earlier by Dole [6].

### *Application of ACS-ACOD method to the MCA*

Reagents were prepared according to the manufacturers' instructions. Assay volumes were then adjusted to suit the capacity of the MCA analytical rotor. For routine analysis, sample (or distilled water as reference) and reagent A (containing ACS, coenzyme A, adenosine triphosphate and 4-aminoantipyrine in 0·05 M phosphate buffer pH 6·9) were added to the inner sample-well using the loader 'sample' and '2nd reagent' settings indicated in table 1 (*a*). Rotors were preincubated for 10 min at 37°C in a hot-air oven, then 160 μl (60% reagent syringe capacity) of reagent B (containing ACOD, POD and 3-methyl-*N*-ethyl-*N*-[6-hydroxyethyl] aniline, MEHA) was added to the outer reagent-well with the loader reprogrammed as in table 1 (*b*). The rotor was analysed using the 'Substrate II' tape with parameters set as shown in table 2, except in some preliminary studies where the general absorbance program was used.

### *Statistics*

Results for the method comparison were evaluated by simple regression analysis and by calculating the mean ± standard deviation (S.D.) difference between values obtained by the two procedures, as recommended by Altman [7].

### Results

### *Optimization of run conditions*

The change in absorbance for aqueous solutions containing 0·1 to 2·5 mmol/litre oleic acid was monitored for 10 min at 550 nm. Maximum colour formation was not reached until 240 s. However, initial reaction rates, particularly for higher concentrations, were very rapid and it was impossible to obtain reliable initial absorbance readings for the assay mixtures. A bichromatic technique was therefore applied using a blanking wavelength of 690 nm, and a reading wavelength of 550 nm at 300 s.

For plasma or serum samples, preincubation at 37°C with reagent A was found to be essential for optimal colour formation

*Table 1. Loader settings for routine analysis of plasma FFA.*

| | FFA detection range (mmol/litre) | Sample volume* | Total sample volume* | Second reagent* | Reagent volume† | Total reagent volume† | Reagent diluent |
|---|---|---|---|---|---|---|---|
| (*a*) | 0·5–2·0 | 4 | 88 | 84 | 0 | 0 | Reagent |
| | 0·1–1·0 | 8 | 92 | 88 | 0 | 0 | Reagent |
| (*b*) | 0·5–2·0 | 0 | 0 | 0 | 60·0 | 64·0 | Reagent |
| | 0·1–1·0 | 0 | 0 | 0 | 60·0 | 64·0 | Reagent |

† = % of 250 $\mu$l capacity syringe volume.

Cuvette 1 (reference) contained 4 or 8 $\mu$l of distilled water as 'sample'.

*Table 2. Analyser parameter settings (Substrate II tape) for plasma FFA analysis.*

| No. | Designation | Setting |
|---|---|---|
| 1 | Factor | 0 |
| 2 | Low normal | Operator definable |
| 3 | Upper normal | Operator definable |
| 4 | Blank wavelength | 8 (690 nm) |
| 5 | Read wavelength | 6 (550 nm) |
| 6 | Delay (s) | 3 |
| 7 | Data interval (s) | 300 |
| 8 | Max. A | 1·0 |
| 9 | Temperature | 1 (30°C) |
| 10 | Start mode | 2 |

with reagent B to occur. However, this final colour reaction was temperature-independent and, for convenience, analyser measurements were taken at 30°C.

### *Linearity and sample volume*

With a 4 $\mu$l sample volume linearity extended to 2 mmol/litre with adequate absorbance change down to 0·5 mmol/litre. Increasing the sample volume to 8 $\mu$l, and modifying the loader settings as indicated in table 1, enhanced the assay sensitivity giving an effective detection range from 0·1 to 1·0 mmol/litre.

*Table 3. Reproducibility of automated FFA determinations in ovine, bovine and human plasma.*

| | Plasma FFA (mmol/litre) | | |
|---|---|---|---|
| | Ovine | Bovine | Human |
| Mean | 0·52 | 0·94 | 0·46 |
| S.D. | 0·021 | 0·032 | 0·010 |
| C.V. (%) | 4·1 | 3·4 | 1·8 |

For each species, figures quoted are the means for the same 10 plasmas run on 10 different occasions over a four-day period.

### *Recovery of FFA and assay precision*

The mean recovery of 0·2, 0·4, 0·8 and 1·0 $\mu$mol of sodium oleate added to 1 ml aliquots of a pooled normal sheep plasma (with a mean FFA concentration of 0·22 mmol/litre) were 98, 104, 105 and 97% respectively ($N = 5$ for each oleate concentration).

Within-run precision was assessed by assaying 18 aliquots of an ovine plasma pool in a single rotor. The mean ± S.D. FFA concentration was 0·530 ± 0·022 mmol/litre giving a coefficient of variation of 4·2%. Reproducibility between runs was measured using the same series of ovine, bovine and human plasmas ($N = 10$ for each species) on 10 separate occasions over a four-day period: the results are shown in table 3. For ruminant plasma, within- and between-run precisions were similar, but only about half that achieved for human plasma with a comparable FFA level.

### Effect of anticoagulants

No differences were found for FFA concentrations determined in serum or plasma containing oxalate/fluoride or EDTA as anticoagulant. However, in the presence of heparin, FFA values were overestimated by about 10%.

### Stability

At room temperature, FFA were unstable in plasma, giving variable and generally decreasing values with an overall loss of approximately 30% in four days. At 4°C the results were more consistent, falling by only about 5% in the first four days after sampling. FFA were stable for at least two weeks at −20°C.

### Comparison with the manual extraction procedure

Normal ovine plasma samples, from a mixed selection of 37 ewes and lambs, were analysed for FFA by both the automated method described and the manual colorimetric techique of Duncombe [1]. The automated method generally gave slightly lower values, the mean ± S.D. percentage difference between estimates made by the two methods being 4·9 ± 11·1. The equation of the regression line obtained was $y = 0{\cdot}0132 + 1{\cdot}05\,x$ with a correlation coefficient ($r$) of 0·959. A similar comparison of 10 human plasmas gave an equation of the form: $y = 0{\cdot}0013 + 1{\cdot}03\,x$, and a correlation coefficient of 0·999.

### Discussion

Reported here, for the first time as far as the authors are aware, is an automated procedure for the specific enzymatic determination of serum and plasma FFA by centrifugal analysis. The method, based on commercially formulated reagents, is rapid (18 tests every 5 min), sensitive (down to 0·1 mmol/litre FFA), reproducible (see table 3), and requires only small reagent volumes (less than 250 $\mu$l/test). Further, the incorporation of within-sample blanking eliminates the need to run separate sample blanks, a time-consuming and expensive necessity for the manual kit procedure. These modifications not only improve the efficiency of FFA determination, but also reduce the cost per test to approximately one tenth of that for the manual enzymatic method.

FFA concentrations determined by the present method showed good agreement with those obtained by an extraction procedure (figure 1), although the former were generally about

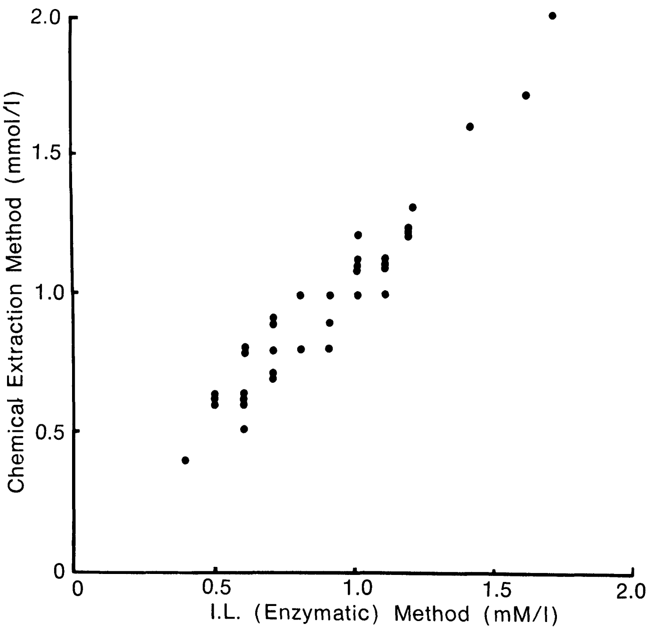

*Figure 1. Comparison between ovine plasma FFA values obtained by manual chemical extraction and automated enzymatic analysis.*

5% lower. These results are consistent with previous comparative studies between enzymatic and extraction-based techniques described by Shimizu *et al.* [2], who attributed the differences to the fact that only about 90% of normal human FFA are within the C6–C18 chain length limits for the ACS used in the enzymatic method.

The sensitivity of the procedure described is sufficient to allow accurate measurement of FFA even in fasting human patients, whose levels are usually between 0·13 and 0·45 mmol/litre (Kushiro *et al.* [8]). As suggested by the manufacturers, heparinized plasma was found to give FFA values which were falsely elevated by about 10%. However, it should be mentioned that Shimizu *et al.* [2] reported that heparin added to human serum had no effect on the estimated FFA concentration.

FFA in ovine plasma were highly unstable at room temperature, but could be preserved for at least 24 h at 4°C. Therefore it is essential that samples should be kept cool after collection and during preparation for assay. When frozen at −20°C, FFA were stable for at least two weeks. Rat plasma FFA have been reported stable for over a week at −15°C, although values rose sharply thereafter (Hron and Menahan [9]). Similar traits have been observed even at ultra-low (−196°C) storage temperatures (Trichopoulou *et al.* [10]; Bergmann *et al.* [11]).

The method described provides a cost-effective routine FFA analysis, which can be practicably applied to large numbers of samples, making it a potentially valuable tool in both a clinical and research context.

### References

1. Duncombe, W. G., *Biochemical Journal*, **88** (1963), 7.
2. Shimizu, S., Inoue, K., Tani, Y. and Yamada, H., *Analytical Biochemistry*, **98** (1979), 341.
3. Mizuno, K., Toyosato, M., Yabumoto, S., Tanimizu, I. and Hirakawa, H., *Analytical Biochemistry*, **108** (1980), 6.
4. Shimizu, S., Tani, Y., Yamada, H., Tabata, M. and Murachi, T., *Analytical Biochemistry*, **107** (1980), 193.
5. Matsubara, C., Nishikawa, Y., Yoshida, Y. and Takamura, K., *Analytical Biochemistry*, **130** (1983), 128.
6. Dole, V. P., *Journal of Clinical Investigation*, **35** (1956), 150.
7. Altman, D. G., *British Medical Journal*, **281** (1980), 1473.
8. Kushiro, H., Takano, K. and Fukui, I., *Japanese Journal of Clinical Pathology*, **15** (1971), 191.
9. Hron, W. T. and Menahan, L. A., *Journal of Lipid Research*, **22** (1981), 377.
10. Trichopoulou, A., Kalaidzidou, C. and Kalandidi, A., *Clinica Chimica Acta*, **69** (1976), 355.
11. Bergmann, S. R., Carlson, E., Dannen, E. and Sobel, B. E., *Clinica Chimica Acta*, **104** (1980), 53.

### Forthcoming papers

In our October–December issue (Vol. 6, No. 3) we will be publishing the following articles:

P. J. Gemperline and R. Megargle—A flexible computer-to-computer protocol for DISNET: a Distributed Instrument System Network.

J. C. W. G. Bink and H. A. Van't Klooster—A microcomputer-based automated curve tracer for accurate digitization of paper-recorded spectra.

M. J. Adams and G. J. Ewen—A microcomputer system for use with an EPR spectrometer.

M. Koupparis and P. Anagstopoulou—An automated microprocessor-based spectrophotometric flow-injection analyser.

R. Stanley—A multi-dimensional approach to analytical science: from invention and prototypes to business on an international scale (1939–1976).

G. A. Harff—The Abbott TDX evaluated for T-uptake.

P. J. Gemperline and R. Megargle—The implementation of a GC/MS data system using DISNET: a Distributed Instrument System Network.

E. F. Legg and M. Sullivan—Turbidimetric analysis on the Hitachi 705 using orosmucoid as a model.

Vol. 7, No. 1 (January–March 1985) will include:

M. Pécs *et al.*—Automatic analyser/computer system for adaptive control of phosphate concentration during fermentation.

M. Legrànd and P. Bolla—A fully automatic apparatus for chemical reactions on the laboratory scale.

J. B. Hemel *et al.*—Critical discussion on a method for derivation of reference limits in clinical chemistry from a patient population.

F. Parri and E. de Majo—Evaluation of an automated haemolytic method for the determination of anti-Streptolysin O antibodies.

